# Investigation of Cranial Bone Changes Indicative of Increased Intracranial Pressure in Diverse Phenotypes of Craniosynostosis

**DOI:** 10.1097/GOX.0000000000006618

**Published:** 2025-03-20

**Authors:** Jasmine Chaij, Jiawei Liu, Brooke French, David Mirsky, Randy C. Miles, Marius George Linguraru, Phuong D. Nguyen, Allyson L. Alexander, Carsten Görg, Antonio R. Porras

**Affiliations:** From the * Department of Pediatric Plastic and Reconstructive Surgery, Children’s Hospital Colorado, Aurora, CO; † Department of Biostatistics and Informatics, Colorado School of Public Health, University of Colorado Anschutz Medical Campus, Aurora, CO; ‡ Department of Radiology, Children’s Hospital Colorado, Aurora, CO; § Department of Radiology, Denver Health, Denver, CO; ¶ Sheikh Zayed Institute for Pediatric Surgical Innovation, Children’s National Hospital, Washington, DC; ∥ Departments of Radiology and Pediatrics, George Washington University School of Medicine and Health Sciences, Washington, DC; ** Department of Pediatric Neurosurgery, Children’s Hospital Colorado, Aurora, CO; †† Departments of Pediatrics, Surgery and Biomedical Informatics, School of Medicine, University of Colorado Anschutz Medical Campus, Aurora, CO.

## Abstract

**Background:**

Despite the clinical importance of identifying increased intracranial pressure (IIP) in children with craniosynostosis (CS), its presence is often uncertain due to limited utilization of invasive measurement methods, inconclusive clinical evaluations, and its variability depending on the CS phenotype. Hence, prevalence reports are highly variable. We previously developed a computational method to identify pediatric chronic IIP of diverse etiology based on subtle cranial thickness and density anomalies quantified from computed tomography (CT) scans. In this study, we evaluate cranial signs of IIP in a large dataset of presurgical CT scans of patients with diverse phenotypes of CS and its prevalence.

**Methods:**

We quantified local cranial thickness and density in the CT scans of 417 patients with diverse phenotypes of CS (age 0–2 y). We used a normative reference of cranial development to quantify cranial bone anomalies in each phenotypic group and compared them with 48 patients with chronic IIP unrelated to CS. We then studied the risk of IIP and its prevalence in each phenotypic group of CS.

**Results:**

Patients with CS presented significant calvarial thickening and bone density decrease compared with normative patients (*P* < 0.001). Similar findings were found in patients with chronic IIP unrelated to CS (*P* > 0.23). Presurgical signs of IIP were more prevalent in patients with Apert syndrome (>74% patients) and nonsyndromic patients with coronal involvement (>30%) compared with other phenotypes (>18%).

**Conclusions:**

Computational evaluation of routinely acquired presurgical CT scans can potentially support the evaluation of IIP in patients with CS.

**Takeaways**

**Question:** Do children with craniosynostosis (CS) present intracranial hypertension before treatment?

**Findings:** We evaluated computed tomography images of 417 children with diverse phenotypes of CS and found that they present similar cranial anomalies to patients with intracranial hypertension unrelated to CS. These were more prevalent in patients with coronal and/or syndromic CS compared with midline CS.

**Meaning:** Disagreements about the prevalence of intracranial hypertension in children with CS are caused by the lack of accepted and reliable noninvasive protocols for its identification. We show the potential of using routinely acquired computed tomography images to support the evaluation of increased intracranial pressure in children with CS.

## INTRODUCTION

Craniosynostosis (CS) is the premature fusion of one or more cranial sutures resulting in restricted expansion of the calvaria perpendicularly to the fused sutures^1^ and compensatory volumetric overdevelopment parallel to them, producing malformations. If untreated, secondary increased intracranial pressure (IIP) can cause detrimental effects, including developmental delay, vision impairment, and/or migraines.^2–6^ Hence, the identification of signs of IIP directly impacts the approach and timing of surgical treatment.^7^

Children with IIP secondary to CS typically display nonspecific and inconsistent^8,9^ clinical symptoms of subjective interpretation^10,11^ with slow or chronic progression.^12^ Because of this variability, there are no accepted clinical protocols to identify IIP.^8,13,14^ Most institutions avoid invasive intracranial measurements or lumbar punctures because of their risks and absence of accepted thresholds to diagnose pediatric IIP.^15–17^ Additionally, radiological findings in computed tomography (CT), including orbital and skull base anomalies, or in magnetic resonance images, including brain lesions, CSF accumulation, sinus thrombosis, and venous anomalies, are variable and often not present in children with chronic IIP.^18,19^ Moreover, evaluation of papilledema has poor sensitivity in children with chronic IIP.^20^

Given the difficulty in diagnosing IIP in patients with CS, prevalence reports are variable. IIP is reported to be present in 1.9%^21^ to 69.4%^22^ of patients with nonsyndromic CS (NSCS) depending on phenotype and age.^7,12,13,23,24^ In patients with syndromic CS (SCS), prevalence of IIP is reported to range from 38%^25^ to 83%.^26,27^ In patients with SCS, the identification of IIP is more relevant, as some institutions delay surgical interventions until IIP has been diagnosed.^26^

Although pediatric radiological studies have reported imaging signs of chronic IIP, they are subtle and variable depending on patient age and sex.^7,12,28–31^ This has prevented the creation of image analysis tools to aid in its diagnosis.^32–35^ Recently, we leveraged a normative statistical model of cranial development to build a machine learning method that could identify bone density and thickness anomalies associated with chronic IIP of diverse etiology unrelated to CS (eg, hydrocephalus, low-grade neoplasms, and pseudotumor cerebri) using standard CT scans.^36^ We also showed that patients with nonsyndromic sagittal CS display similar cranial anomalies to patients with chronic IIP unrelated to CS. In this work, we leverage our previous method to evaluate cranial anomalies associated with IIP, identify its prevalence in patients with diverse phenotypes of NSCS and SCS using their standard presurgical CT scans, and show its potential to support the clinical evaluation of CS.

## MATERIALS AND METHODS

### Data Description

Four retrospective CT image datasets of children younger than 10 years, collected between 2005 and 2022, were obtained after internal review board approval at the University of Colorado Anschutz Medical Campus (protocol no. 20-1563) and Children’s National Hospital (protocol no. 3792). Dataset A includes CT scans of 1018 normative patients (539 boys, age 3.08 ± 3.02 y, range 0–10 y), which were used in previous study^36^ to create a normative model of pediatric cranial development that accounts for age, sex, and image resolution.^37^ Dataset B contains CT scans of 48 patients with confirmed chronic IIP (23 boys, 25 girls, age 4.23 ± 3.13 y, range 0–10 y) of diverse etiology unrelated to CS (ie, hydrocephalus, hemorrhage, idiopathic IIP, arachnoid cysts, and intracranial neoplasms). These patients were followed up clinically until symptomatic, qualitative, and quantitative signs of IIP (average symptom duration 2.48 ± 2.91 mo) prompted diagnostic confirmation and treatment.^36^ This dataset was used to establish a quantitative reference of cranial anomalies in children with chronic IIP. Additional details about these datasets can be found in a previous study.^36^

For the current study, we curated 2 additional CT image datasets of patients with CS before treatment. Dataset C contains cross-sectional presurgical CT scans of 403 patients (266 boys, 137 girls, age 4.68 ± 3.96 mo, range 0–2 y) with single-suture NSCS. The dataset includes 79 patients with metopic (MC), 242 patients with sagittal (SC), 63 patients with unicoronal (UCC), and 19 patients with bicoronal (BCC) NSCS. Patients with lambdoid NSCS were excluded because of an insufficient number of patients with this rare phenotype of CS.^38^ Patients with history of craniofacial surgery, known genetic or hereditary conditions affecting skull development, or other known comorbidities were excluded to eliminate factors that could affect cranial density and/or thickness. Dataset D includes presurgical CT scans of 14 patients with SCS and genetic confirmation of Apert syndrome (AS) (7 boys, 7 girls, age 3.00 ± 3.12 mo, range 0–2 y). Five of these patients had a second presurgical scan available at the age of 7.69 ± 5.64 months. All images were acquired as part of standard clinical protocols. To minimize variability associated with partial volume effects and image resolution, images with in-plane resolution larger than 0.5 mm or slice thickness larger than 1.5 mm were excluded from all datasets. All dataset details are summarized in Table 1.

**Table 1.** Details of Datasets A (Normative Subjects), B (Patients With Chronic IIP), C (Patients With Nonsyndromic Single Suture CS), and D (Patients With AS)

	Dataset A	Dataset B	Dataset C	Dataset D
Sample size				
No. patients/images	1018/1018	48/48	403/403	14/19
Age, y				
Mean ± SD (first/second)	3.08 ± 3.02	4.23 ± 3.13	0.39 ± 0.33	0.25 ± 0.26/0.35 ± 0.36
Sex				
Male	539 (52.95%)	23 (47.92%)	266 (66.00%)	7/8 (50.00%/42.11%)
Female	479 (47.05%)	25 (52.08%)	137 (34.00%)	7/11 (50.00%/57.89%)
Original CT image voxel volume (before resampling)				
Mean ± SD (first/second), mm^3^	0.105 ± 0.057	0.095 ± 0.030	0.087 ± 0.045	0.056 ± 0.017/0.065 ± 0.015
Fused suture (dataset C)				
MC	NA	NA	79 (19.60%)	NA
SC	NA	NA	242 (60.05%)	NA
UCC	NA	NA	63 (15.63%)	NA
BCC	NA	NA	19 (4.71%)	NA

NA, not applicable.

### Quantification of Cranial Bone Anomalies

We used previous methods to quantify cranial thickness and density from each CT image. All images were resampled to a uniform in-plane resolution of 0.5 mm and slice thickness of 1.5 mm using linear interpolation to reduce variability associated with partial volume effects.^36^ We used our public deep learning model to segment the calvaria, label the cranial bones,^39^ and create a standard anatomical representation with local correspondences between subjects guided by the location of the cranial base and sutures.^40^ All segmentations were inspected and manually corrected if needed using ITK-SNAP. Cranial bone thickness (in mm) and density (in Hounsfield units) were calculated at every location of the calvaria and averaged both for the entire calvaria and separately within each bone as described in prior work.^36^ In the current work, we extended previous linear regression to evaluate thickness and density differences among datasets A, B, C, and D, accounting for age, sex, and the original CT image voxel as


$$\begin{array}{l} \begin{array}{l} Y_{i}^{l}=\beta_{0}^{l}+\beta_{1}^{l}*Age_{i}+\beta_{2}^{l}*Sex_{i}+\beta_{3}^{l}*V_{i}+\beta_{4}^{l}*IIP_{i}+\;\\ \;\;\;\;\;\;\;\;\;\;\;\beta_{5}^{l}*MC_{i}+\beta_{6}^{l}*SC_{i}+\;\beta_{7}^{l}*UCC_{i}+\beta_{8}^{l}*BCC_{i}+\beta_{9}^{l}*AS_{i},\; \end{array} \end{array}$$ (1)

where $Y_{i}^{l}$ represents the cranial bone thickness or density of patient i at cranial location l; $\beta_{j}^{l},\;j=\left\{0,\cdots ,9\right\}$ are the regression parameters for each cranial location l; Age_*i*_ represents the age of patient i; $V_{i}$ represents the original image voxel volume in millimeter cube; and Sex_*i*_ is a binary indicator of patient sex (1 for male, 0 for female). IIP_*i*_ indicates if the patient belongs to the group of patients with chronic IIP unrelated to CS (dataset B). MC_*i*_, SC_*i*_, UCC_*i*_, and BCC_*i*_ are binary indicators representing if the patient belongs to the nonsyndromic group (dataset C) and is diagnosed with MC, SC, UCC, or BCC, respectively. Finally, AS_*i*_ is a binary indicator indicating if the patient presents AS (dataset D). IIP_*i*_, MC_*i*_, SC_*i*_, UCC_*i*_, BCC_*i*_, and AS_*i*_ are mutually exclusive to enable statistical group comparisons. Note that all diagnostic binary indicators set to zero represent normative subjects (dataset A). Statistical differences across patient groups were evaluated using *t* tests.

### Investigation of Cranial Bone Signs of Chronic IIP in Patients With CS

In a previous study,^36^ we developed a logistic regression classifier to automatically identify cranial bone anomalies indicative of chronic IIP based on the sex-, age- and voxel volume–adjusted cranial bone density quantified for datasets A and B. In the current work, we used a prior model to evaluate the probabilistic risk of patients with CS (datasets C and D) presenting chronic IIP and its prevalence based on their sex-, age- and voxel volume–adjusted values, similar to previous work.^36^ We evaluated 2 thresholds on the probabilistic output of our model: (1) the threshold that provides the optimal binary classification accuracy (evaluated via cross-validation in prior work^36^) between datasets A and B based on the Youden index (YI), which balances sensitivity and specificity and (2) the threshold that provides at least 95% specificity, to investigate the utility of our model for routine screening using CT images acquired as part of standard clinical practice for patients with CS. Finally, we quantified the Spearman rank correlation between the probabilistic risk of IIP and age for patients with CS to study if older children who were not operated on are at higher risk of IIP as suggested in previous studies.^41^

## RESULTS

### Quantification of Cranial Bone Anomalies

Table 2 presents the differences in cranial bone thickness and density among normative subjects (dataset A), patients with chronic IIP unrelated to CS (dataset B), patients with NSCS (dataset C), and patients with AS (dataset D) from our regression model, accounting for the effects of age, sex and CT image voxel volume. Because patients with left and right UCC present asymmetrical anomalies, their bone thickness and density anomalies are aggregated separately in Table 3.

**Table 2.** Mean and SD of the Local Differences in Cranial Bone Thickness (in mm) and Density (in HU) Between Patient Groups: Normative, Patients With IIP Unrelated to CS (IIP), Patients With Nonsyndromic SC, MC, BCC CS, and Patients With CS Secondary to AS

	Differences in Thickness (mm)			Differences in Bone Density (HU)		
	Mean	SD	*P*	Mean	SD	*P*
Frontal bones						
IIP versus normative	0.06	0.09	0.51	−174.65	27.68	<0.001*
MC versus normative	0.51	0.05	<0.001*	−155.80	14.81	<0.001*
SC versus normative	0.47	0.03	<0.001*	−140.24	9.81	<0.001*
BCC versus normative	0.61	0.09	<0.001*	−161.63	27.87	<0.001*
AS versus normative	0.39	0.09	<0.001*	−255.63	27.88	<0.001*
MC versus IIP	0.45	0.10	<0.001*	18.86	30.85	0.89
SC versus IIP	0.41	0.10	<0.001*	34.42	28.76	0.50
BCC versus IIP	0.55	0.13	<0.001*	13.03	38.68	0.98
AS versus IIP	0.33	0.13	0.04†	−80.97	38.79	0.10
Parietal bones						
IIP versus normative	−0.04	0.08	0.62	−175.35	24.62	<0.001*
MC versus normative	0.37	0.04	<0.001*	−134.10	13.17	<0.001*
SC versus normative	0.37	0.03	<0.001*	−165.51	8.73	<0.001*
BCC versus normative	0.40	0.08	<0.001*	−125.55	24.79	<0.001*
AS versus normative	0.24	0.08	0.001†	−197.99	24.80	<0.001*
MC versus IIP	0.41	0.08	<0.001*	41.25	27.44	0.31
SC versus IIP	0.41	0.08	<0.001*	9.84	25.58	0.97
BCC versus IIP	0.44	0.11	<0.001*	49.80	34.40	0.34
AS versus IIP	0.28	0.11	0.02†	−22.65	34.50	0.86
Occipital bones						
IIP versus normative	0.18	0.09	0.04†	−120.31	24.88	<0.001*
MC versus normative	0.35	0.05	<0.001*	−94.44	13.31	<0.001*
SC versus normative	0.35	0.03	<0.001*	−122.30	8.82	<0.001*
BCC versus normative	0.51	0.09	<0.001*	−95.02	25.05	<0.001*
AS versus normative	0.33	0.09	<0.001*	−109.07	25.06	<0.001*
MC versus IIP	0.17	0.10	0.21	25.87	27.73	0.68
SC versus IIP	0.17	0.09	0.15	−1.99	25.85	1.00
BCC versus IIP	0.33	0.12	0.02†	25.29	34.77	0.82
AS versus IIP	0.15	0.12	0.49	11.24	34.86	0.99
Average in calvaria						
IIP versus normative	0.04	0.08	0.63	−163.73	23.87	<0.001*
MC versus normative	0.41	0.04	<0.001*	−132.92	12.77	<0.001*
SC versus normative	0.40	0.03	<0.001*	−149.60	8.46	<0.001*
BCC versus normative	0.50	0.08	<0.001*	−131.84	24.03	<0.001*
AS versus normative	0.31	0.08	<0.001*	−196.47	24.04	<0.001*
MC versus IIP	0.37	0.09	<0.001*	30.81	26.60	0.52
SC versus IIP	0.36	0.08	<0.001*	14.14	24.80	0.91
BCC versus IIP	0.46	0.11	<0.001*	31.89	33.35	0.66
AS versus IIP	0.27	0.11	0.03†	−32.74	33.44	0.65

Note that the effects of age, sex, and CT image voxel resolution are removed using equation 1.

* Statistical significance for *P* < 0.01.

† Statistical significance for *P* < 0.05.

HU, Hounsfield unit.

**Table 3.** Mean and SD of the Local Differences in Cranial Bone Thickness (mm) and Density (HU) Between Patient Groups: Normative, Patients With IIP Unrelated to CS (IIP), and Patients With Nonsyndromic UCC

	Difference in Thickness (mm)			Difference in Bone Density (HU)		
	Mean	SD	*P*	Mean	SD	*P*
Frontal bones on the side of the fused coronal sutures						
UCC versus normative	0.37	0.06	<0.001*	−143.80	17.04	<0.001*
UCC versus IIP	0.30	0.11	0.005†	32.21	33.21	0.33
Frontal bones on the side of the open coronal sutures						
UCC versus normative	0.49	0.55	<0.001*	−138.67	16.89	<0.001*
UCC versus IIP	0.42	0.11	<0.001*	36.79	32.92	0.26
Parietal bones on the side of the fused coronal sutures						
UCC versus normative	0.26	0.04	<0.001*	−141.59	15.31	<0.001*
UCC versus IIP	0.29	0.09	0.001†	34.66	29.85	0.25
Parietal bones on the side of the open coronal sutures						
UCC versus normative	0.40	0.05	<0.001*	−135.94	15.14	<0.001*
UCC versus IIP	0.44	0.09	<0.001*	39.71	29.51	0.18
Occipital bone						
UCC versus normative	0.31	0.05	<0.001*	−90.68	14.52	<0.001*
UCC versus IIP	0.11	0.10	0.25	33.51	28.30	0.24
Average in calvaria						
UCC versus normative	0.37	0.05	<0.001*	−130.96	14.69	<0.001*
UCC versus IIP	0.32	0.09	<0.001*	34.06	28.64	0.23

Note that the effects of age, sex, and CT image voxel resolution are removed using equation 1.

* Statistical significance for *P* < 0.01.

† Statistical significance for *P* < 0.05.

HU, Hounsfield unit.

All patients with CS presented global cranial bone thickening compared with normative subjects (*P* < 0.001) and patients with chronic IIP (*P* = 0.03). Locally, the frontal and parietal bones were thicker in all patients with NSCS compared with normative (*P* < 0.001) and patients with chronic IIP (*P* < 0.005). In patients with AS, although a similar thickening was found compared with normative subjects (*P* < 0.001), this was only significant in the frontal and parietal bones (*P* ≤ 0.04) compared with patients with chronic IIP. Although all patients with CS had significant occipital thickening compared with normative subjects (*P* < 0.001), only patients with BCC showed significant thickening compared with patients with chronic IIP (*P* = 0.02).

**Fig. 1.**
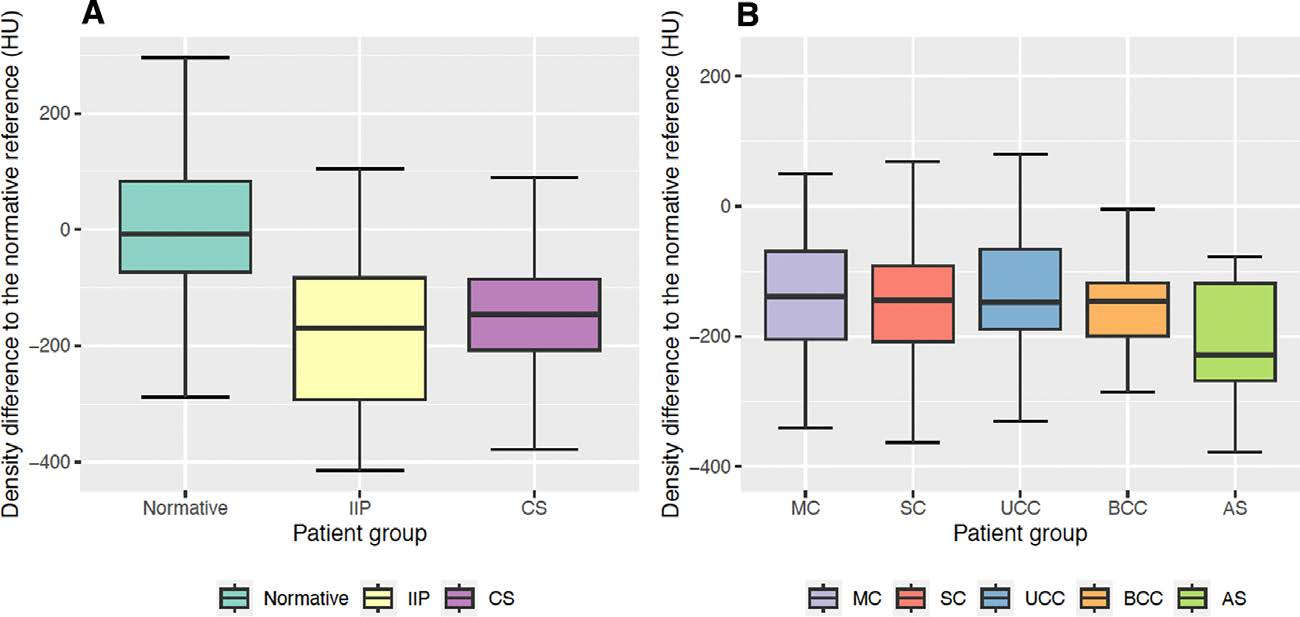
Cranial bone density anomalies. A, Distributions of the cranial bone density differences between the average normative statistical reference and different patient groups: normative subjects (dataset A), patients with chronic IIP unrelated to CS (dataset B), and patients with CS (datasets C and D). B, Distributions of the cranial bone density differences between the average normative statistical reference and patients with diverse phenotypes of CS: MC, SC, UCC, BCC, and SCS secondary to AS. Note that the normative statistical reference is adjusted for age, sex, and image resolution. HU, Hounsfield unit.

Separately, all patients with CS presented a significant bone density decrease compared with normative subjects (*P* < 0.001), both globally and at each cranial bone. This decrease was not different to findings in patients with chronic IIP unrelated to CS, either globally (*P* = 0.23) or locally (*P* ≥ 0.1). Figure 1A presents the distribution of bone density differences among the age-, sex-, and voxel size–specific normative average reference from equation 1, and datasets A, B, C, and D. Figure 1B presents these distributions in patients with different phenotypes of CS in datasets C (NSCS) and D (AS).

### Cranial Bone Indications of IIP in Patients With CS

**Fig. 2.**
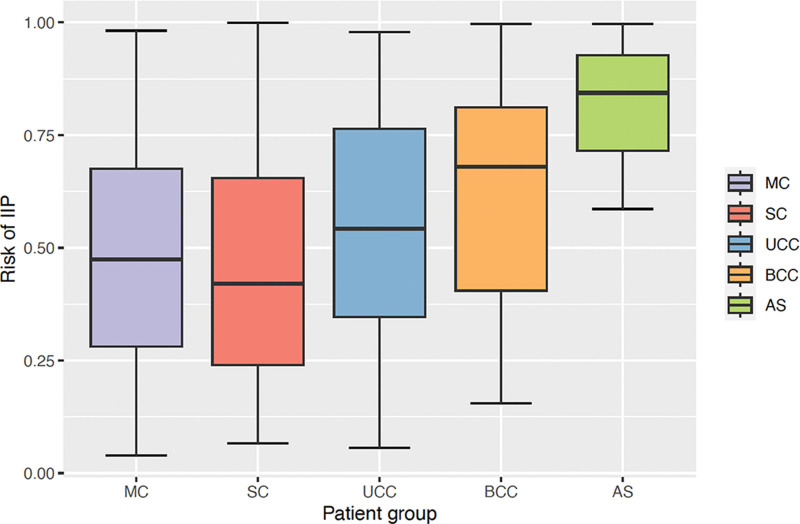
Distributions of risk of IIP in patients with diverse phenotypes of CS: MC, SC, UCC, BCC, and SCS secondary to AS.

Figure 2 presents the probabilistic risk of IIP for datasets C and D quantified using our logistic regression classifier.^36^ We observed significantly higher probability of IIP in patients with MC (48.60 ± 24.29%), SC (45.79 ± 25.42%), UCC (55.14 ± 23.98%), BCC (60.80 ± 25.76%), and AS (75.81 ± 23.67%), compared with normative subjects (30.83 ± 20.54%; *P* < 0.001 with Mann-Whitney *U* test and Bonferroni correction). We also observed higher risk of IIP in patients with chronic IIP unrelated to CS (66.70 ± 28.37%) compared with patients with MC (*P* = 0.003) and SC (*P* < 0.001). No significant differences were found between patients with confirmed chronic IIP unrelated to CS, and patients with UCC (*P* = 0.14), BCC (*P* = 1.00), or AS (*P* = 1.00).

The receiving operator characteristic curve showing model performance for different probability thresholds was presented in prior work.^36^ The IIP probability risk threshold that maximized YI was 54%, which provided a sensitivity and specificity of 81.25% and 84.97%, respectively, evaluated through cross-validation on datasets A and B.^36^ The threshold that provided at least 95% specificity was 73%, which yielded 56.25% sensitivity and 95.09% specificity. Table 4 presents the estimated prevalence of IIP for our patients with CS in datasets C and D using these 2 thresholds. Patients with AS present the highest estimated prevalence of IIP (84.21% for YI and 73.68% for the 95% specificity threshold), followed by BCC (63.16% for YI and 42.11% for the 95% specificity threshold), UCC (52.38% for YI and 30.16% for the 95% specificity threshold), MC (43.04% for YI and 17.72% for the 95% specificity threshold), and SC (36.89% for YI and 18.03% for the 95% specificity threshold). Finally, we found that the risk of IIP was positively correlated with age (Spearman correlation coefficient 0.30; *P* < 0.001).

**Table 4.** Probabilistic Risk of IIP Quantified for Patient Groups With Different Phenotypes of CS, and Proportion of Patients Presenting High Risk of Chronic IIP Evaluated Using 2 Different Thresholds: the Threshold That Maximizes the Performance of the Model Identifying IIP Using the YI, and the Threshold That Provides 95% Specificity

	Dataset C				Dataset D
	MC	SC	UCC	BCC	AS
Probabilistic risk of IIP (mean ± SD), %	48.60 ± 24.29	45.79 ± 25.42	55.14 ± 23.98	60.80 ± 25.76	75.81 ± 23.67
% Patients with IIP using YI threshold	43.04	36.89	52.38	63.16	84.21
% Patients with IIP using 95% specificity threshold	17.72	18.03	30.16	42.11	73.68

## DISCUSSION

We studied cranial bone anomalies in patients with diverse phenotypes of CS, accounting for age, sex, and image resolution. Our previous work^36^ showed that these findings can be utilized to identify signs of IIP from standard CT scans, and our current study shows the potential of our framework to support the evaluation of IIP in patients with CS.

Our results show that all patients with CS (datasets C and D) exhibit thicker and less dense cranial bone than normative subjects, similarly to patients with chronic IIP unrelated to CS (dataset B). Notably, there was no difference between the cranial bone density of patients with CS and patients with chronic IIP unrelated to CS. These findings align with previous studies^42,43^ and may be explained by cranial remodeling and increased bone porosity to compensate for IIP.^44,45^ Cranial osteocytes respond to IIP, absorbing denser cortical bone and remodeling the inner diploic layer.^46,47^ Although adult IIP studies report cranial bone thinning,^33,48^ they do not reflect pediatric bone remodeling capabilities.^49^ Studies have shown that nonacute IIP causes bone thickening as opposed to the bone thinning observed in acute IIP,^43,50^ with imaging studies showing thickened bone around the fused sutures, focal bone thickening,^51^ and osteoblast-mediated thickening of the outer cranial surface.^52^ Hence, bone density loss may be the only systematic bone anomaly in children with chronic IIP.

There are large disagreements about the presence of IIP in patients with CS. Although some practitioners would discard signs of IIP based on clinical evaluation in most patients with NSCS, works in the literature show a prevalence of IIP in these patients up to 69.4%,^22^ which increases to 83%^26,27^ in patients with SCS. We leveraged our quantitative model to calculate the probabilistic risk of IIP in patients with diverse phenotypes of CS based on their associated cranial bone anomalies,^36^ and we estimated the prevalence of IIP before surgical treatment in different phenotypes of CS. Our results show a significantly higher risk of IIP in all patients with CS compared with normative. We found that children with midline suture fusion presented lower risk of IIP compared with patients with coronal suture fusions, who presented a similarly high risk of IIP than patients with IIP unrelated to CS. Children undergo a faster expansion in the anteroposterior direction during early life^37,40^ because of faster coronal suture growth,^53^ which explains a decreasing cephalic index with age.^54^ We hypothesize that premature coronal suture fusion and its associated volumetric constraints translate faster to an increase of IIP. Additionally, the coronal arch has a more complex composition than the sagittal arch^55–57^ and its developmental disruptions may produce a chained effect in other surrounding structures contributing to increased incidence of IIP.^56,58^ Moreover, patients with UCC and BCC are more likely to present undiagnosed genetic syndromes such as Muenke syndrome,^59^ which have variable expressivity^59–61^ and higher risk of IIP.^62^ Indeed, all our patients with SCS secondary to AS presented BCC and higher rates of IIP compared with other groups. The complex clinical sequelae accompanying AS (eg, sleep apnea, venous hypertension) and the potential involvement of minor suture fusions^58,63,64^ may also contribute to their higher risk of IIP.^65^

To explore the potential of our methods for screening purposes using standard images, we studied the prevalence of IIP using a risk probability threshold that provided 95% specificity, which indicated that at least 17.72%, 18.03%, 30.16%, 42.11%, and 73.68% of patients with MC, SC, UCC, BCC, and AS, respectively, present signs of IIP before treatment. Although some institutions prefer to not operate on very young children with CS because of increased risks,^66,67^ the early identification of IIP typically prompts treatment^7^ because of its association with worse neurodevelopmental outcomes^3–5^ and higher morbidity risk.^68^ Our results show that some patients presented signs of IIP at the time of diagnosis and suggest that they could have benefitted from earlier interventions. This is also supported by the significant correlation found between the risk of IIP and age. Hence, once prospectively validated, our method could be used with the acquired standard CT images to enable the earlier detection of IIP to prompt more timely treatments and improve patient outcomes.

One limitation of this study is the sample size of patients with AS compared with patients with NSC, although our sample size was large enough to find significant bone thickening and density loss compared with normative subjects. This was due to our strict inclusion criteria of genetic diagnostic confirmation and the use of only preoperative CT images to avoid biases and variability in our statistical analysis. We believe that the inclusion of these patients was important to show that cranial anomalies were present in both syndromic and nonsyndromic forms of CS. We did not include patients with other genetic diagnoses because of insufficient sample size.

Another limitation is related to data variability within our CT image datasets. We used strict inclusion criteria to minimize potential comorbidities affecting cranial development and reduce variability associated with image resolution and acquisition protocol. In addition, our method accounted for age, sex, and image voxel size (ie, partial volume effects). Although our approach showed great performance on large retrospective multi-institutional datasets that were not acquired for this research, uncontrolled biases (eg, nutrition, medication, epigenetics) may potentially affect our study and a prospective study using variable imaging protocols and diverse patient groups could provide additional insight about the robustness of our methods to data variability.

Finally, because this study is retrospective and invasive measurements of pressure are not part of standard protocols in patients with CS, we could not confirm the presence or absence of IIP in these patients. Instead, we utilized independent datasets of normative subjects and patients with confirmed IIP unrelated to CS to demonstrate that patients with CS present similar cranial bone anomalies than other patients with IIP of diverse etiology, and we provided quantitative evidence that these can be systematically quantified from standard CT images. We believe that a prospective study obtaining diagnostic confirmation is the next step to validate the clinical application of our method.

## CONCLUSIONS

We presented a method to quantitatively and systematically evaluate the risk of IIP in patients with diverse phenotypes of CS based on cranial bone anomalies identified from their routinely acquired CT images. Although a prospective validation study will be required before clinical translation, we showed the potential of our approach to support the evaluation of signs of IIP in patients with CS, which is essential to optimize their surgical management and improve outcomes. Additionally, our computational framework could help mitigate the challenges of time-consuming and increasingly expensive additional imaging studies that are often inconclusive and acquired for children with CS suspected to present IIP.

## DISCLOSURES

The authors have no financial interest to declare in relation to the content of this article. Research reported in this publication was supported by the National Institute of Dental and Craniofacial Research (NICDR) of the National Institutes of Health under award number R21DE031824. This work was also supported by NICDR under grant number R00DE027993 and by NIH/NCATS Colorado CTSA under grant number UM1 TR004933. The content is solely the responsibility of the authors and does not necessarily represent the official views of the National Institutes of Health.
